# An examination of the demographic predictors of adolescent breakfast consumption, content, and context

**DOI:** 10.1186/1471-2458-14-264

**Published:** 2014-03-20

**Authors:** Barbara Mullan, Cara Wong, Emily Kothe, Kathleen O’Moore, Kristen Pickles, Kirby Sainsbury

**Affiliations:** 1 School of Psychology, The University of Sydney, Sydney, NSW, Australia; 2 School of Psychology and Speech Pathology, Curtin University, Perth, WA, Australia; 3 School of Psychology, Deakin University, Melbourne, VIC, 3125, Australia

**Keywords:** Breakfast, Australia, England, Diet, Nutrition

## Abstract

**Background:**

Breakfast consumption is important to health; however, adolescents often skip breakfast, and an increased understanding of the breakfast consumption patterns of adolescents is needed. The purpose of this study was to identify the predictors of breakfast eating, including the content and context, in an adolescent sample from Australia and England.

**Methods:**

Four-hundred and eighty-one students completed an online questionnaire measuring breakfast skipping, and breakfast content (what was eaten) and context (who they ate with, involvement in preparation). Logistic regression was conducted to investigate the predictors of skipping breakfast, breakfast context, and consumption of the ten most commonly consumed foods. Chi-square analyses were used to examine differences in breakfast content according to context.

**Results:**

Most students (88%) had consumed breakfast on the day of the survey; breakfast skipping was more common in England (18%) than in Australia (8%). Country, gender, socioeconomic status, and body mass index (BMI) were all predictors of breakfast content and context. Whether adolescents ate with others and/or were involved in breakfast preparation predicted the content of breakfast consumed.

**Conclusions:**

This study provides a comprehensive examination of the factors underlying breakfast consumption (content and context) and has important implications for the development of evidence-based interventions to improve rates of breakfast consumption and the quality of food consumed amongst adolescents.

## Background

The consumption of a healthy breakfast is important to health [1]. Specifically, eating breakfast is associated with improved nutrient intake [2-5] and lower body mass index [2,3,5,6]. The impact of regular breakfast consumption on overall daily energy intake is, however, unclear, with discrepancies likely due to differences in the type of food consumed at breakfast [7], as well as whether breakfast consumption is related to regular healthy eating or, in contrast, associated with a pattern of eating more throughout the day. Regular breakfast consumption is also associated with reduced stress, depression, and emotional distress [8]. Amongst young people, eating breakfast correlates with improved school attendance, whilst skipping breakfast interferes with memory and attention [9].

Nutrition-related chronic diseases (e.g., type 2 diabetes, cardiovascular disease, and obesity) are increasing in the adolescent population [10,11], and may be due in part to skipping breakfast. In a large sample of 13–16 year old Dutch adolescents, breakfast skipping was more strongly associated with being overweight than physical inactivity [6]. Further, after controlling for age, gender, lifestyle factors, and socio-demographics, individuals who skipped breakfast throughout childhood and adulthood were found to have higher fasting insulin levels, higher cholesterol, and greater waist circumferences than those who ate breakfast [12].

Breakfast is the most commonly skipped meal in adolescence [1], with declines in both the frequency [13] and the quality of breakfast as children age [14]. Illustrative of reduced quality, adolescents in one study consumed soft drinks and desserts in place of nutritious breakfast foods [2], and less than 5% of teenagers in another study were classified as consuming a nutritionally balanced breakfast [15]. Evidence suggests that the health benefits of breakfast consumption may be partially determined by factors such as food type [16]; portion size [17]; processed/unprocessed food [18]; glycaemic index [19]; kilojoules [20]; and variety of foods [15].

Research suggests that there are differences in adolescents’ breakfast patterns – including both the frequency and quality of breakfast – across different countries. For example, the preferred breakfast for adolescents (aged 11–14) from North Italy was bakery items [21]; in the Netherlands the most commonly consumed breakfast items come from the grain and dairy food groups [22]; in Cape Town, adolescents (aged 11–17) commonly eat white bread, chocolate and potato crisps [23]; in the USA and Scotland, ready-to-eat cereals are preferred [24,25]; while in Australia, the most commonly consumed breakfast items were cereals and bread [20]. The interpretation of this data is, however, hampered by the lack of a universal definition of breakfast and the use of different methodologies [26]. For example, forced response questionnaires tend to classify consumption using broad categories such as cereals (which include ready-to-eat cereals and hot cereals such as porridge) and breads (which include muffins, crumpets, and bread rolls), making it difficult to determine healthy or unhealthy patterns [20]. Several interventions designed to increase breakfast consumption have been conducted in countries such as Australia, the UK, and the USA [27]; however, the overall lack of efficacy highlights the need to increase understanding of the factors underlying breakfast consumption within these countries in order to design successful interventions.

In addition to the content of breakfast, research has suggested that the context in which breakfast is consumed (encompassing whether or not the adolescent is involved in preparing their own breakfast, and who they eat breakfast with, as well as other family-related factors) may be an important determinant of both breakfast skipping and quality. For example, it has been found that parental breakfast eating is the most significant factor associated with adolescent breakfast eating [28,29], suggesting that parents may play a pivotal role in influencing breakfast choices, likely via establishing norms around breakfast eating as well as controlling the availability and preparation of food. Similarly, irregular breakfast consumption was associated with family structure (single vs. both parents) [3,29,30], an increased number of siblings, and having a mother who worked [30,31]. Despite the apparent importance of parental influence on breakfast consumption, one study found that only 15% of parents monitored and encouraged children’s breakfast eating [21]. Adolescent boys who received positive encouragement to eat a healthy diet were more likely to regularly consume breakfast, as were adolescent girls who perceived their friends to eat healthily, suggesting that peers are also influential in breakfast consumption patterns [32]. The frequency of family meals in general has also been positively associated with healthier food choices in adolescence [33].

The aim of the current study was to investigate the demographic predictors of breakfast skipping amongst adolescents (aged 11–18 years), as well as to examine any differences in breakfast content patterns according to whether adolescents ate breakfast alone or with others, and whether they were involved in preparing their own breakfast or not. Although previous research has assessed both the content [20-25] and context [28-33] of breakfast consumption, this has tended to be in isolation (that is, assess content or context, not both) and few have looked specifically at the interaction between these factors. Further, as previously stated, previous research has been limited by the use of questionnaire designs that make it difficult to determine whether specific breakfast choices are indeed healthy or unhealthy. This study therefore employed a comprehensive purpose-designed online food diary to capture a wider variety of food items (including different food types, brands, and portion sizes) than has previously been possible, as well as to enable the number of kilojoules consumed to be calculated.

## Method

### Design and procedure

A cross-sectional, between-subjects design was employed. The recruitment strategy has been described elsewhere [34]. Briefly, schools in both urban and rural areas and from a range of socio-economic backgrounds in Australia and England were approached for recruitment. Twenty-five schools agreed to participate; however, external factors (e.g., time constraints) led to a high dropout rate. Within the participating schools (n = 9), classroom teachers were nominated to oversee the online data collection, and adolescents in the classes of each selected teacher on the day of data collection were approached to participate during their class time. Student participation was voluntary and informed consent was obtained prior to data collection. The majority of consenting schools nominated students aged 14–15 (n = 490), predominantly due to the higher workload of older students. This study was conducted in accordance with the Helsinki Declaration, and approval was obtained from the University of Sydney Human Research Ethics Committee (10637), the State Education Research Approval Process (2008099) as well as from the individual schools.

The primary outcome variables of interest were breakfast content (i.e., what was consumed for breakfast; including food type and portion size) and context (i.e., whether the adolescent was involved in their own breakfast preparation and who they ate breakfast with) and these were assessed using a comprehensive purpose-designed online questionnaire (described in detail in the next section). Breakfast was defined as ‘any food consumed within two hours of waking’.

### Measures

Participants completed demographic information including gender, age, ethnicity, living situation, and maternal and paternal occupation (used to estimate socio-economic status), and self-reported their height and weight [to enable calculation of BMI; [35]. A novel online food diary was developed for the study based on relevant literature [20,36,37] including two systematic reviews [26,27] conducted as part of extensive formative research prior to the current study, and feedback from a pilot study which examined breakfast content in university students [38]. One common limitation identified in the systematic reviews that may have impacted the results and interpretation of many previous breakfast studies was the tendency to categorise participant’s breakfast choices as healthy/unhealthy or good/bad while failing to recognise variations in nutritional quality between food items within the same broad category, failing to account for portion size, adopting arbitrary criteria regarding the distinction between healthy and unhealthy [26,27]. For example, one study classified students’ breakfasts as ‘good quality’ only if they contained foods from the dairy, cereal, and fruit groups. This is problematic as although it is indeed recommended that individuals consume certain amounts of each of these foods, an overall diet may still be healthy even if these foods are not consumed at breakfast time. The purpose-designed questionnaire used here was therefore developed to overcome these limitations by including a broad range of food categories, and rather than classifying breakfasts as healthy or unhealthy based on endorsement of specific categories, more detailed questions were then included to allow specification of the actual foods and amounts eaten and for manual calculation of the calories consumed. The specific food categories included in the questionnaire were selected based on the categories typically used in similar research as well as drawing on the findings of the previously mentioned pilot study.

Initially, students indicated whether they had consumed breakfast that morning (current day breakfast consumption), and, if so, they then worked through the online questionnaire. Participants who indicated that they had not consumed breakfast that day were asked to list reasons (free text response). Participants who had consumed breakfast were asked to indicate, from a pre-defined list (categories derived from previous research described above), which categories of food and drink their breakfast had included: bread/toast; bakery goods; breakfast bar; cereal; cooked/hot breakfast; fresh fruit; yoghurt; sweets/chocolate; food from previous meal; fast food; and other. Beverage categories included water; soft drink/cordial; smoothie; milk/milkshake; fruit juice; and hot drink. For each category endorsed, participants were then asked to indicate the type (combination of free text response and forced choice; e.g., within bakery goods: muffin, croissant, bread roll; type of milk: full fat, low fat, skim, soy, other) and brand of the item (free text response with examples provided; e.g., Kellogs, Just Juice); whether they added anything to the item (either forced choice or free text; e.g., jam, or sugar); and the portion size of each food and drink item consumed.

In order to obtain an accurate indication of portion size (to allow manual computation of kilojoules), for each food/drink item a photo of an average portion was included. A measurement scale appropriate to the item was also included (e.g., a cup measure and/or a scale in centimetres). Participants were asked to indicate whether they ate less than/about the same as/more than what was in the picture. Average portion sizes for relevant items were taken from the Dietary Guidelines for Australian Adults [37] and the UK Food Standards Agency [39]. For example, the average portion size for both cereal and beverages was one cup; the portion size for bread/toast was two slices; for bakery items the portion size was a medium bread roll/bakery item. Participants then indicated with whom they consumed breakfast and whether they prepared their own breakfast (breakfast context). The questionnaire was administered using Quask software; for further details including the precise questions and response options, as well as for examples of the pictures presented for portion size, please see Additional file 1.

### Data analysis

Kilojoules consumed at breakfast by each participant were manually computed over several weeks with great precision using the detailed information on breakfast content and portion sizes collected in the online questionnaire (kilocalories = kilojoules/4.184). Independent samples t-tests and chi-square analyses were used to determine whether the Australian and English samples differed according to demographic variables. Subsequently, a logistic regression analysis was used to predict breakfast skipping. Amongst participants who had eaten breakfast, a multiple linear regression analysis was used to examine predictors of kilojoules consumed. Having identified the ten most commonly consumed food categories, a series of logistic regression analyses were then conducted to determine the significant predictors of consuming each food category (coded as no/yes). Logistic regression analyses were also used to determine the significant predictors of breakfast context: firstly, whether the participants ate breakfast alone or with other people, and secondly, whether the participants prepared their own breakfast or whether it was prepared by somebody else. In each of the analyses the independent variables were gender, age, country, BMI (underweight, normal weight, overweight, obese), and socio-economic status (SES: low, medium, high).

Amongst the participants who had eaten breakfast, independent samples t-tests were used to compare differences in the number of kilojoules consumed between those who ate breakfast alone and those who ate with others; and between those who prepared their own breakfast and those whose breakfast was prepared for them. Chi-square tests of independence were similarly used to examine differences in breakfast content by context.

## Results

### Demographics

A total of 481 adolescents from four Australian (n = 321; Brisbane, Dubbo, Bathurst, and Hurstville) and five English schools (n = 160; Oxfordshire, Worcestershire, Gloucestershire, Yorkshire and Hampshire) participated in the study. Participants ranged from 11 to 18 years old (*M* = 13.97, *SD* = 1.17), and the majority were female (69.6%; male: 30.4%). Ninety-eight percent of participants lived with their parents, and 72.4% identified as Caucasian. The majority of the sample (68.4%) was from high SES backgrounds (see Table 1). Several significant differences were found between respondents from the two countries; the Australian sample was older (*t*_479_ = 6.87, *p* < .001), had more females (*χ*^2^ = 43.78, *p* < .001) and had more students from high SES (*χ*^2^ = 28.88, *p* < .001) compared to the English sample. Significantly more Australian students were normal weight compared to the UK sample (*χ*^2^ = 15.93, *p* < .001), while the opposite pattern was observed for overweight (*χ*^2^ = 10.19, *p* < .01) and obese (*χ*^2^ = 6.37, *p* < .05).

**Table 1:** Demographics of the Australian and English participants

	**Australia**		**England**	
**Age**	**14.17 years (SD = 1.1)**		**13.46 years (SD = 0.98)**	
	**N**	**(%)**	**n**	**(%)**
Gender				
Males	66	20.6	80	50
Females	255	79.4	80	50
Ethnicity				
Caucasian	237	73.8	113	70.6
Asian	28	8.7	-	-
Black/African	1	0.3	5	3.1
Middle Eastern	3	0.9	-	-
Other	52	16.2	42	26.3
SES				
High	246	76.6	84	52.5
Middle	60	18.7	62	38.8
Low	15	4.7	10	6.3

Note: SES was calculated from father’s occupation; 4 English participants did not provide an indication of paternal education, resulting in missing data for this variable (% do not add up to 100).

### Skipping breakfast

Overall, 12% of the sample reported skipping breakfast on the morning of the study. English students were significantly more likely to skip breakfast than Australian students (OR = 0.33; *p* = .002). There were no differences in breakfast skipping according to age, gender, BMI or SES. Among those who skipped breakfast, the reasons cited included not having time or being too busy (42.9%); not being hungry in the morning (24.3%); not enjoying breakfast (15.7%); and weight control (4.3%).

### Breakfast content

Amongst the students who had consumed breakfast on the day of data collection, gender was the only significant predictor of kilojoule consumption at breakfast (*β* = −.21; *p* < .001); with males (mean kj consumption = 1969) eating significantly more kilojoules at breakfast than females (mean kj consumption = 1387). There were no differences in kilojoule consumption at breakfast according to country, age, BMI or SES (all *p* > .05).

As can be seen in Table 2, the most commonly consumed breakfast item was cereal, with almost half the sample (n = 225) consuming cereal alone or with other items for breakfast. The most popular cereals included weetbix/weetabix (n = 54), cornflakes (n = 24) and cocopops (n = 15), and the majority added milk to their cereal (n = 210; full fat: n = 65; low fat: n = 62; skim: n = 65; soy: n = 1; other: n = 17). The next most frequently eaten item was bread/toast (n = 152), including white bread (n = 74), which was more common than brown or wholemeal bread (n = 46), and grain (n = 9) or fruit bread (n = 9). Most added something to it (n = 136), with butter being the most common (n = 60), followed by vegemite/marmite (n = 39). Fresh fruit was eaten by 18.3% (n = 98) of the sample, many of whom endorsed several types of fruit, with apples (n = 23), bananas (n = 23), and mango (n = 16) being the most common choices. The most common drinks consumed were fruit juice (29.2%) and water (22.8%), followed by milk (17.3%; full fat: n = 22; low fat: n = 22; skim: n = 17; soy: n = 1), and a hot drink such as coffee, tea or hot chocolate (13.8%).

**Table 2:** Frequency of consumption of different food items

**Breakfast food item**	**Total %**	
	**n**	**%**
Cereal	225	45.8
Bread/Toast	152	31.0
Fruit juice	138	28.1
Water	123	25.1
Fresh fruit	98	20
Milk	88	17.9
Hot drink	59	12.0
Bakery goods	38	7.7
Cooked breakfast	40	8.1
Yoghurt	34	6.9
Smoothie	17	3.5
Cereal bar	14	2.9
Chocolate	13	2.6
Soft drink or cordial	11	2.2
Leftovers	8	1.6
Fast food	3	0.6
Sweets/Lollies	2	0.4
Other	41	8.4
**Context**		
Eats alone	174	35.4
Helps to prepare own breakfast	261	53.2

Note: statistical comparisons were conducted for the ten most commonly eaten food categories.

An examination of the 10 most commonly consumed foods (denoted by #) indicated a significant gender effect for cereal (#1) consumption, whereby boys were 3.22 times more likely than girls to eat cereal for breakfast (*p* < .001). Overweight children were less likely to consume cereal compared to normal weight children (OR = 0.39, *p* = .01); and children in the medium SES category were less likely to consume cereal than low SES children (OR = 0.19, *p* < .001).

Regarding fruit juice (#3) consumption, English adolescents were more likely than Australian adolescents to consume fruit juice at breakfast (OR = 1.76, *p* = .017). The opposite effect was found for water (#4; OR = 0.33, *p* < .001), fresh fruit (#5; OR = 0.47, *p* = 0.16), and consumption of a cooked breakfast (#9; OR = 0.33, *p =* .021). Adolescents in the medium SES and high SES groups were both significantly less likely than children in the low SES group to consume milk with breakfast (OR = 0.35, *p* = .021 and OR = 0.30, *p* = .005 respectively). There were no effects of gender, country, age, BMI category or SES category on the likelihood of consuming a breakfast that included toast (#2), a hot drink (#7), bakery items (#8) or yoghurt (#10; all *p* > .05).

### Breakfast context

Information about the context of breakfast consumption is presented in Table 2. The only significant predictors of breakfast preparation were age and BMI category. Specifically older adolescents were more likely to be involved in breakfast preparation than younger adolescents (OR = 1.56, *p* = .03); while adolescents falling in the obese BMI category were less likely than those in the normal weight BMI category to prepare their own breakfast (OR = 0.21, *p* = .025). There were no differences in breakfast preparation according to gender, country, or SES category (all *p* > .05). Regarding eating with other people, older adolescents were less likely to eat breakfast with others compared to younger adolescents (OR = 0.60, *p* = .004), as were English adolescents compared to Australian (OR = 0.36, *p* = .019). There were no differences according to gender, BMI category, or SES category (all *p* > .05).

### Contextual differences in breakfast content

There was no difference in the total number of kilojoules consumed at breakfast between those who prepared breakfast and those who did not (*p* > .05). There was, however, a significant difference in total kilojoules consumed between those who ate alone and those who ate with others, such that eating with others resulted in consumption of an average additional 335kj compared to participants who ate alone (*t* = 2.59, *p* = .01). As can be seen in Table 3, those who ate with others were more likely to consume toast, fruit juice, and a cooked breakfast than those who ate breakfast alone. Those who made their own breakfast were more likely to consume cereal and less likely to consume hot drinks, bakery items, and cooked breakfasts than those did not.

**Table 3:** Differences in food content by food context

**Breakfast food item**	**Eating situation**		**Difference**	**Helps make breakfast**		**Difference**
	**With others %**	**Alone %**	***p***	**No %**	**Yes %**	***p***
Cereal	44.0	43.1	.503	27.0	48.0	.**001**
Bread/Toast	35.0	22.4	**.012**	31.1	28.0	.601
Fruit juice	34.18	21.8	**.012**	23.0	29.1	.297
Water	28.0	20.0	.098	20.3	24.5	.447
Fresh fruit	25.3	19.0	.163	21.6	22.2	.912
Milk	19.0	19.5	.899	20.3	18.8	.773
Hot drink	9.5	10.9	.669	17.6	8.8	**.032**
Bakery goods	10.1	6.9	.290	17.6	5.7	**.001**
Yoghurt	8.2	6.3	.503	6.7	7.3	.880
Cooked breakfast	11.4	5.2	**.038**	20.3	5.0	**<.001**
Cereal bar	1.3	3.4		1.4	2.3	
Smoothie	1.9	4.0		1.6	3.4	
Soft drink or Cordial	1.9	2.3		2.7	2.0	
Chocolate	0.6	1.1		0	1.1	
Leftovers	1.3	1.7		1.4	1.5	
Other	7.0	6.9		4.0	7.7	

Note: statistical comparisons were conducted for the ten most commonly eaten food categories; bolded p values indicate those that were significant at the level of p < .05.

## Discussion

The overall aim of this study was to examine the breakfast patterns, including content and context, of adolescents from two countries. This was achieved using a detailed, purpose-designed food diary containing a wider variety of food categories than has previously been used. Further, the focus on breakfast consumption on the day of data collection served to reduce memory biases that have potentially impacted the findings of previous research. The rates of skipping breakfast (12%) and the observation that this was more common in England than Australia are largely consistent with previous data [20,40,41]. Importantly, however, the current study is the first to demonstrate this difference using the same methods across the two countries, and highlights the importance of multi-country recruitment methods.

Eight percent of Australian adolescents skipped breakfast. This is lower than previous findings where 10-12% of Australian adolescents were found to regularly skip breakfast [20,40]. This slight discrepancy may reflect improved breakfast consumption patterns since 1995, when the most recent Australia data was collected [20]. Alternatively, the more comprehensive methodology used in the current study may have impacted the results. Regarding the English sample, the present rate of breakfast skipping (18%) was consistent with UK data, which has previously found that 19% of adolescents aged 11–16 years regularly missed breakfast [41].

### Breakfast content

Ready-to-eat cereal was the most commonly consumed breakfast food, followed by bread products and fruit juice. This is comparable to results from a systematic review where it was found that ready-to-eat cereals and dairy foods were the most commonly consumed breakfast items, followed by fruit and fruit juice, and bread products [26]. Despite some concerns surrounding the nutritional benefits of certain cereals and bread products (outlined below), the Australian and English adolescents in the current sample were generally making better food choices at breakfast compared to previous research, which has revealed high consumption of ‘unhealthy foods’ such as sweets/chocolate and bakery goods [21,23].

It has been suggested that consuming a breakfast that includes cereal is beneficial to overall nutrient intake, as some cereals (e.g., weetbix/weetabix) are indeed low in fat, good sources of complex carbohydrates, fortified with essential nutrients, and high in dietary fibre [2]. In contrast, there are concerns about the nutritional quality of other ready-to-eat cereals (e.g., cocopops) based on their high sugar content and lack of nutrients [42]. In the current study the most popular ready-to-eat cereals were weetbix/weetabix, and cornflakes, both of which have relatively low sugar content, although cocopops, which is higher in sugar, was also frequently consumed [43]. Thus, while eating certain types of cereals for breakfast may have health benefits, the consumption of high sugar cereals and the addition of sugar to cereal, also have important implications for glycaemic control [44], and may therefore be of concern for a proportion of the current sample.

Bread was another commonly consumed breakfast item, with a strong preference for white bread. This is consistent with previous research showing that most adolescents and young adults prefer white bread to brown bread [4,45]. These findings do, however, raise similar concerns to the above, as wholegrain products contain more nutrients and fibre and are lower on the glycaemic index, therefore sustaining satiety for longer compared to white products. Consumption of wholegrain products has also been linked to reductions in obesity, cardiovascular disease and type II diabetes [46].

There was a significant effect of SES on the consumption of cereal and milk, such that adolescents from medium SES backgrounds were less likely to consume cereal and milk than adolescents from low SES backgrounds. This finding may reflect the relatively lower expense of cereals compared to other breakfast items (e.g., fresh fruit or bakery items). Adolescents in the normal weight category were more likely to consume cereal than overweight adolescents, indirectly supporting the assertion that eating certain types of cereals may help to reduce the ill health effects (including obesity) associated with poor nutrition, particularly at breakfast. This is consistent with previous research where it was found that children and adolescents who ate cereal for breakfast had lower BMI and waist circumferences, as well as lower rates of obesity, than those who skipped breakfast [3].

#### Breakfast context

In the current study, two-thirds of adolescents indicated that they ate breakfast with another person/people, most commonly members of their family, and approximately half the participants were involved in preparing their own breakfasts. Obese children were less likely than normal weight children to prepare their own breakfasts, perhaps reflecting the desire of parents of obese children to control their children’s breakfast consumption and specifically the nutritional/calorie content. Previous research has found that parental breakfast eating is the most significant factor associated with adolescent breakfast eating [28]. It is therefore of some concern that only a small proportion of the adolescents in the current study (18% of breakfast eaters) reported eating breakfast with their parents or with their whole family, while a large proportion (35% of breakfast eaters) ate breakfast alone. Older adolescents in particular were more likely to eat alone than younger adolescents. When combined with evidence that rates of breakfast consumption tend to decline with age, this finding suggests that the continued involvement of family in the breakfast meal regardless of age is likely to be helpful.

Several differences in breakfast content were found according to whether participants ate alone or with others, as well as whether they were involved in breakfast preparation. Adolescents who ate breakfast alone were less likely to consume bread/toast, fruit juice, and a cooked breakfast than those who ate with other people, while adolescents who prepared their own breakfasts were more likely to consume cereal and less likely to consume hot drinks, bakery items and a cooked breakfast. This pattern of findings is consistent with the relatively greater effort involved in preparing these food types for one person and the time constraints potentially involved in breakfast preparation/eating for this age group. Finally, participants from England were more likely to eat breakfast by themselves than those in Australia, although the reason for this is unclear. Research on the motivators behind breakfast consumption has found that subjective norm (perceptions of the expectation of others for you to perform a behaviour, as well as whether or not other people are performing the behaviour), is a significant predictor of breakfast consumption [36,38], with these findings supporting a link between the presence of other people and breakfast content. The impact of this on overall daily energy intake, however, remains unclear. That is, consuming more at breakfast may be indicative of a healthy diet whereby energy consumed throughout the day is normally distributed or skewed towards the morning [47], or alternatively, a generalised pattern of overeating which continues throughout the day leading to excessive energy intake. Future interventions to improve breakfast consumption may benefit from the inclusion of strategies to encourage parental presence and involvement at breakfast time.

### Strengths and limitations

This study had several potential limitations that should be considered when interpreting the results. Firstly, breakfast consumption was measured on a single occasion on the day of data collection. Although this decision was based on several considerations including the reduced likelihood of recall bias compared to measures of a typical day’s breakfast [26], and measurement at a single time point being less burdensome than measures of breakfast consumption taken across multiple days, it is possible that such a measure may not be representative of actual breakfast consumption patterns captured using weeklong measures. Further, the survey focused on breakfast consumption only and as such it is unclear whether those participants who ate fewer kilojoules went on to eat more or less over the course of the day. It is therefore not possible to draw firm conclusions about how healthy the participants’ overall diets were based on this information. Secondly, the skew towards higher SES observed in this study may have led to an overestimation of breakfast eating as high SES has previously been associated with breakfast eating rather than breakfast skipping [48]. Given that the aim of the study was to investigate the factors associated with the content and context of breakfast consumption (rather than breakfast skipping per se), however, this was not seen as detrimental. The rate of breakfast skipping for English students and the significant difference in breakfast skipping between Australia and England was comparable to previous studies, suggesting that the uneven distribution of SES across the sample did not have a major impact on the results. Finally, despite the large sample size, participants were recruited from a limited number of schools, meaning that the findings may not be generalisable to the wider adolescent population.

This study also had several strengths including the recruitment of adolescents from two different countries and the use of detailed food diaries for data collection, which enabled the calculation of kilojoules as well as a more comprehensive analysis of breakfast content than previous studies that have been limited to food categories rather than specific items.

### Implications and future directions

This study is the first to provide a comprehensive and simultaneous exploration of the content and context of breakfast consumed by adolescents in Australia and England. Importantly, the focus on factors associated with breakfast consumption/skipping in addition to the specific content and context means that these results have the potential to inform interventions aimed not only at increasing rates of breakfast consumption but also at improving the overall quality of breakfasts. Whilst the majority of adolescents did eat breakfast, a strong preference for ready-to eat cereals and white bread was observed, with fewer students eating foods from dairy and fruit groups. Future exploratory studies may consider measuring breakfast consumption over a longer period to ensure the data is representative and generalisable, as well as measuring energy intake across the course of the day rather than only at breakfast.

Future breakfast interventions could explore ways to change eating behaviour at both the individual level (e.g., encouraging healthy individual food choices) and the societal level (e.g., focusing on educating parents). For example, encouraging adolescents and their parents to substitute high sugar cereals with those that are not only lower in sugar but also have additional health benefits (e.g., fibre, nutrients) – an approach adopted in the current Australian “swap it don’t stop it” campaign [49]. Further improvements in breakfast content that should be targeted in interventions include increasing consumption of non-white grains and fresh fruit at breakfast, as well as tailoring nutrition advice based on differences in age, SES, and BMI status to ensure adolescents from all demographic groups are equipped to make healthy breakfast choices. Finally, encouraging the continuing involvement of parents in their adolescent’s breakfast preparation and consumption may also help to improve the quality of breakfasts in this age group.

## Conclusions

Overall, these findings represent an important advancement over previous research by not only detailing the rates of breakfast skipping (which has been a major focus of previous studies) but also examining what adolescents are actually eating for breakfast (i.e., the content) and the interaction between this and the context in which breakfast is being consumed. The results have important implications for future intervention design.

## Competing interest

The authors declare that they have no conflict of interest.

## Authors’ contributions

BM was involved in conceptualizing, generating and co-ordination of the project, and also drafted the manuscript. CW collected data and analyzed the results. EK assisted with the conceptualization of the project and decided on the statistical analyses. KO’M constructed the questionnaires and collected data. KP assisted with the statistical analyses and collected data. KS assisted in drafting the manuscript. All authors read drafts of the manuscript and provided comments. All authors read and approved the final manuscript.

## Pre-publication history

The pre-publication history for this paper can be accessed here:

http://www.biomedcentral.com/1471-2458/14/264/prepub

## Supplementary Material

Additional file 1 Breakfast Eating Questionnaire (content and context). Click here for file
