# Supplementary material for: An examination of the demographic predictors of adolescent breakfast consumption, content, and context
Source: BMC Public Health. 2014 Mar 20;14:264. doi: 10.1186/1471-2458-14-264 (PMC4000053; doi:10.1186/1471-2458-14-264)
Supplement: Additional file 1 — Breakfast Eating Questionnaire (content and context). [file 1471-2458-14-264-S1.docx]

**Additional file 1. Breakfast eating**

Did you eat breakfast this morning? Yes (continue below)

No (automatically redirected to later questions)

Where did you eat breakfast this morning?

- At home
- On the way to school – please specify where
- At school
- Other (e.g., a friend’s house) – please specify where

Please select ALL the items that you had for breakfast this morning?

- Cereal (e.g., cornflakes, muesli, sultana bran)
- Cereal bar
- Bread/toast
- Bakery goods (e.g., cake, muffin, croissant)
- Cooked/hot breakfast (e.g., bacon and eggs)
- Fresh fruit
- Yoghurt
- Chocolate
- Sweets/lollies other than chocolate
- Food from a previous meal (e.g., pizza, chicken and vegetables, pasta)
- Fast food (e.g., McDonalds, Hungry Jacks)
- Cup of water
- Cup of milk or milkshake
- Fruit juice
- Smoothie
- Hot drink (e.g., tea, coffee, hot chocolate)
- Soft drink or cordial (sweetened/flavoured water)
- Other (an item not listed above)

*If yes to cereal:*

What was the type of cereal? (e.g., Kellogg's cornflakes, Kellogg's rice bubbles)

Did you have milk on your cereal? (Yes/No)

If yes, was it:

- Full fat
- Low fat
- Skim
- Soy
- Other – please specify

Did you add anything else to your cereal (e.g., sugar, honey)? (Yes/No) If yes, please specify

How much cereal did you have?


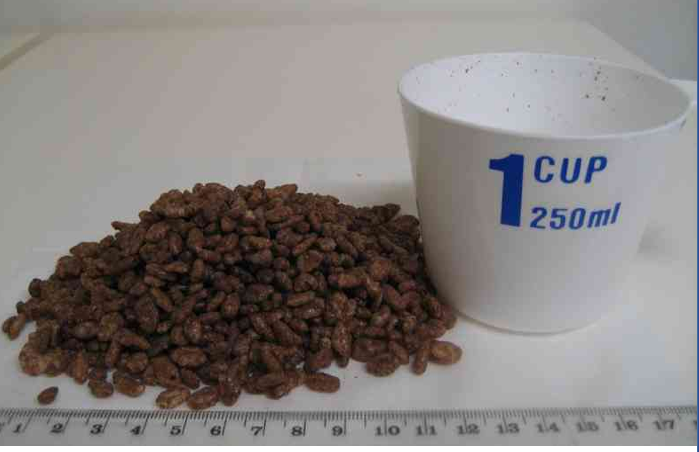


- Less than above (< 1 cup)
- About the same (1 cup)
- More than above (> 1 cup)

*If yes to cereal bar*

What was the brand of the cereal bar (e.g., Kellogs)?

What was the type of cereal bar (e.g., Special K, Rice Bubbles)?

About how many cereal bars did you have?

- 1 bar
- 2 bars
- More than 2 bars

*If yes to bread/toast*

About how many slices did you have?

What was the type of bread that you had (e.g., white, brown, rye, wholemeal, fruit bread)?

Did you have anything on your bread/toast?

If yes, did you have:

- Butter
- Peanut butter
- Vegemite/marmite
- Jam
- Chocolate spread
- Other – please specify

*If yes to bakery goods*

What was the type of bakery good?

- Bread roll
- Cake
- Muffin
- Croissant
- Other – please specify

Did you have anything on your bakery goods?

If yes, did you have:

- Butter
- Jam
- Sugar or honey
- Other – please specify

Please tick how much of the following bread or bakery goods you consumed at breakfast. The picture below is one average portion (tick all that apply).


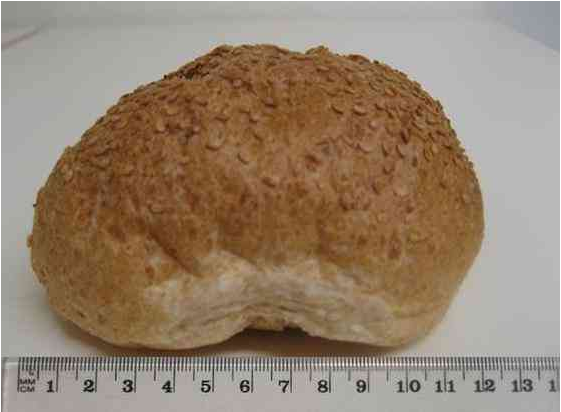


|  | One portion | Two portions | More than two portions |
| --- | --- | --- | --- |
| Bread roll |  |  |  |
| Croissant |  |  |  |
| Muffin |  |  |  |
| Cake |  |  |  |
| Other |  |  |  |

*If yes to fresh fruit*

What was the type of fruit (e.g., orange, strawberries, fresh fruit salad)?

Did you have anything else on your fresh fruit?

If yes, did you have:

- Low fat yoghurt
- Full fat yoghurt
- Sugar or honey
- Other – please specify

If you had large fruit (e.g., apple, orange, melon), how much did you have?


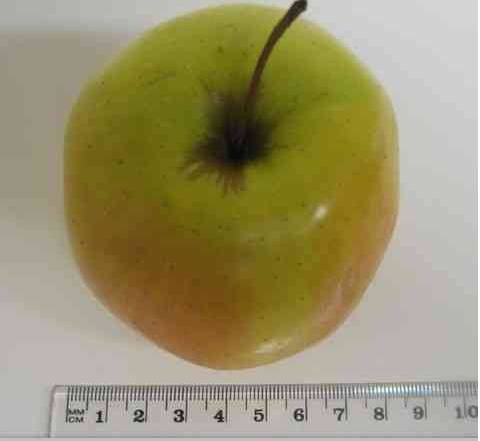


- Less than above
- About the same as above (e.g., one apple)
- More than above
- Did not have large fruit

If you had medium sized fruit (e.g., plum, fig, mandarin), how much did you have?


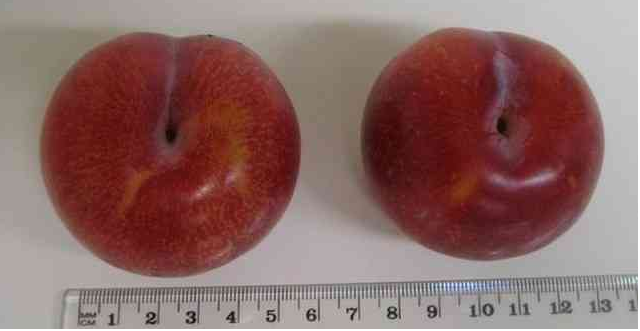


- Less than above
- About the same as above (e.g., two plums)
- More than above
- Did not have medium fruit

If you had small sized fruit (e.g., grapes, berries), how much did you have?


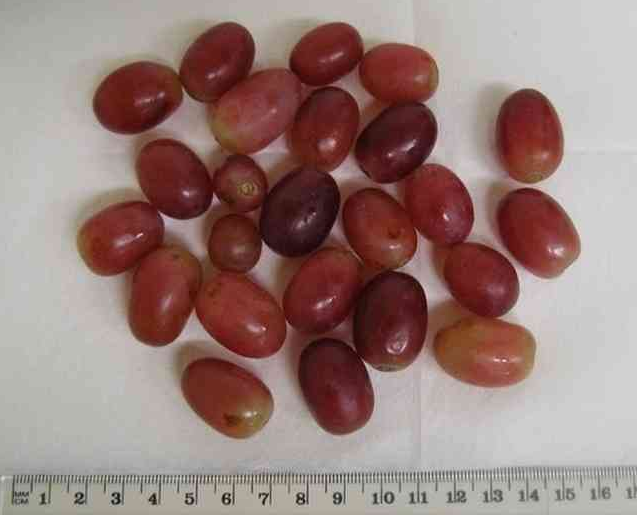


- Less than above
- About the same as above (e.g., two handfuls of grapes)
- More than above
- Did not have small fruit

*If yes to yoghurt*

What was the type of yoghurt (please specify the brand and type; e.g., Yoplait low fat strawberry)

Did you have anything else with your yoghurt?

If yes, did you have:

- Fresh fruit
- Nuts
- Sugar or honey
- Other – please specify

How much yoghurt did you have?


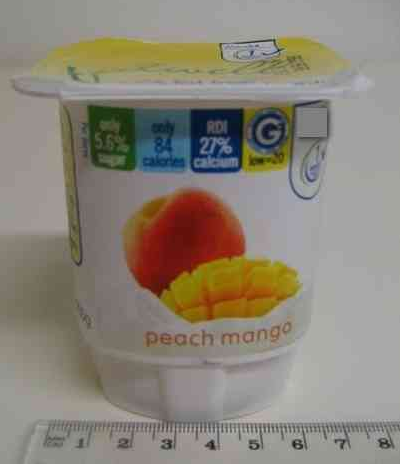


- Less than above (< one tub)
- About the same as above (one tub)
- More than above (> one tub)

*If yes to chocolate*

Was it:

- Milk chocolate
- Dark chocolate
- White chocolate
- Other – please specify

What was the type of chocolate (e.g., Mars Bar, Hersheys chocolate block)

How much chocolate did you have?


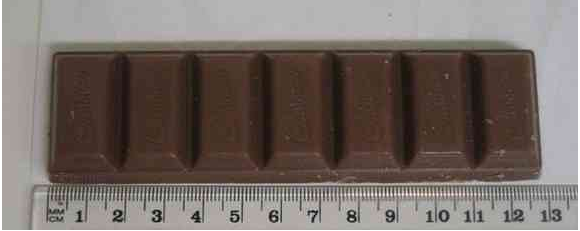


- Less than above (< one 55g bar)
- About the same as above (one 55g bar)
- More than above (> one 55g bar)

*If yes to sweets/lollies*

What was the type of sweet (e.g., lollipop, jellybabies, pick ‘n’ mix, jellybeans)?

How many sweets did you have?


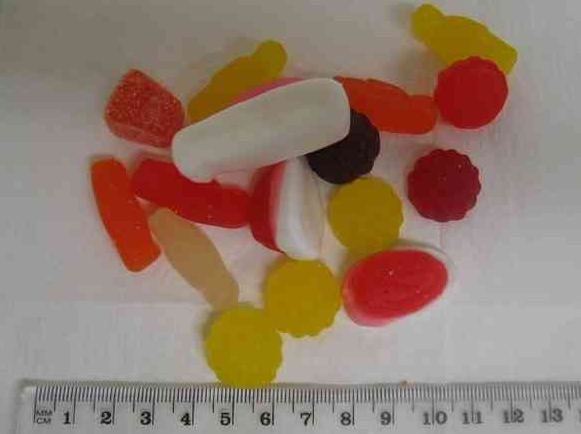


- Less than above
- About the same as above
- More than above

*If yes to fast food*

What was the type of food you had (e.g., McDonalds, Hungry Jacks)?

Select the items of fast food you had and then write in how much of that item you had (e.g., one cheeseburger with small fries):

| **Item** | **Portion size** |
| --- | --- |
| Burger |  |
| Pancakes |  |
| Grilled sandwich |  |
| Hashbrown |  |
| Fries |  |
| Fruit |  |
| Yoghurt |  |
| Sauce (e.g., tomato, barbeque) |  |
| Other (state food and quantity) |  |

*If yes to water*

What was the type of water?

- Filtered
- Tap
- Bottled
- Tank
- Other – please specify

*If yes to fruit juice*

What was the brand of the juice (e.g., Just Juice, Original Juice Company)?

What was the type of juice?

- Juice you squeezed
- Bottled
- Other – please specify

*If yes to smoothie*

What was the type of smoothie?

- Fresh fruit
- Packaged
- Other – please specify

Did you add anything to your smoothie (e.g., honey, chocolate flavour)?

If yes, please specify

*If yes to a hot drink*

What was the type of hot drink?

- Tea
- Coffee
- Hot chocolate
- Other – please specify

Did you have milk in your hot drink?

If yes, was it:

- Full fat
- Low fat
- Skim
- Soy
- Other – please specify

Did you add anything else (e.g., sugar, honey)?

If yes, please specify

*If yes to soft drink/cordial*

Did you have a soft drink this morning?

What was the type of soft drink?

Did you have cordial (sweetened water) this morning?

What was the type of cordial (e.g., Cottee’s orange cordial)?

Please tick how much of the following drinks you consumed at breakfast (tick all that apply)


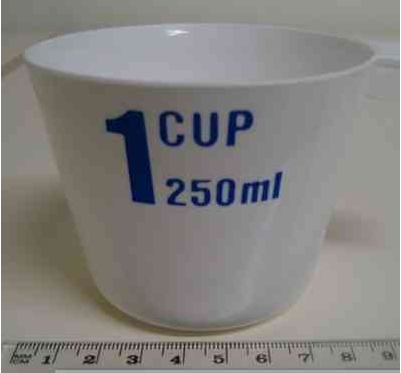


| **Drink** | **One cup** | **Two cups** | **More than two cups** |
| --- | --- | --- | --- |
| Water |  |  |  |
| Milk |  |  |  |
| Juice |  |  |  |
| Smoothie |  |  |  |
| Hot drink |  |  |  |
| Soft drink/cordial |  |  |  |
| Other |  |  |  |

Is there anything else that you had for breakfast that was not listed (Yes/No)?

If yes, please specify the type and quantity of any other items in as much detail as possible (e.g., a cup of almonds, a chocolate coated vanilla ice cream, 2 lemonade icy poles, rice, noodles, pancakes).

*If did not eat breakfast*

For our purposes, breakfast eating is defined as any food or beverage taken during the period between waking and the commencement of morning classes (approximately 9am).

If you did not eat breakfast this morning, please state why.

*Context*

Who prepares your breakfast (e.g., self, mother, sibling)?

Who do you eat breakfast with most of the time (e.g., alone, mother, sibling)?
